# How to build resiliency in autistic individuals: an implication to advance mental health

**DOI:** 10.1186/s40359-024-01916-1

**Published:** 2024-08-01

**Authors:** Parisa Ghanouni, Rebeccah Raphael, Liam Seaker, Amanda Casey

**Affiliations:** 1 Department of Occupational Therapy, Dalhousie University, PO Box 15000, Halifax, NS B3H 4R2 Canada; 2 Department of Human Kinetics, St. Francis Xavier University, Antigonish, Canada

**Keywords:** Autism, Coping strategies, Mental health, Resiliency, Teaching

## Abstract

**Introduction:**

Individuals on the autism spectrum (ASD) often experience poor mental health and coping strategies compared to their peers due to social exclusion and co-occurring conditions. Resiliency has been identified as a key factor in preventing adverse outcomes and promoting mental health. Therefore, it is important to determine what strategies can be used to build resiliency among autistic individuals. The current paper is one of the first studies that aims to collect information from autistic individuals and their caregivers on potential strategies to enhance resiliency.

**Methods:**

We interviewed 18 participants from various provinces in Canada, comprising of 13 autistic individuals and 5 parents. We used thematic analysis to identify patterns in the data.

**Results:**

Thematic analysis revealed three themes to indicate strategies that could be used to enhance resiliency, including: (a) self-reliant strategies, (b) using community-based facilities, and (c) contextual and individual characteristics.

**Conclusion:**

Although the body of literature on resiliency is evolving, this paper provides a unique perspective as it is one of the few studies that considers the experiences of individuals on the spectrum. In addition, this study focuses on identifying and describing specific strategies that can be used to enhance resiliency and mental health, which consequently can help address the existing gaps in knowledge and practice.

**Supplementary Information:**

The online version contains supplementary material available at 10.1186/s40359-024-01916-1.

## Introduction

Mental health is a subjective state of wellbeing, where individuals recognize their skills and abilities to deal with life stressors. It is incomplete to talk about mental health without addressing resiliency, which is the ability to bounce back from negative experiences. Resiliency can not only protect individuals when facing mental health conditions, but also, it can help individuals cope with conditions that increase the risk of mental health, such as traumas or being bullied. Among prevalent disorders, autism symptoms often begin in early childhood and continue into adulthood, which may require lifelong mental health supports [1, 2]. Autistic individuals often have co-occurring mental health and physical conditions, including anxiety or psychological distress [3, 4]. For example, the rate of anxiety disorders amongst autistic individuals lies between 40 and 84%, whereas the prevalence amongst other neurodivergent individuals ranges from 15 to 20% [5, 6]. Due to challenges faced, autistic individuals often require continued holistic support services, ranging from occasional assistance to daily support [7]. However, some individuals may maintain independence by coping with the situation and positive adaptations through their resiliency [8–10].

Resiliency has been generally defined as one’s ability to respond to challenging circumstances and to bounce back from adversity [11]. An individual’s resiliency is determined by balancing attributes that decrease or increase the likelihood of positive outcomes in the face of adversity [12, 13]. Such attributes are frequently referred to as risk and protective factors [13]. Individual factors such as hardiness, extroversion, self-efficacy, openness to change, self-esteem, self-understanding, problem-solving skills, and social competence can advance resiliency. In addition, environmental factors (e.g., social networks, connectedness and cohesion, available and perceived familial and community support, and quality close peer relationships) can also have a positive impact on resiliency [14–17]. Given that there is a lack of evidence on what resiliency means or how it looks like in the autistic community, resiliency could look different among them compared to what it means for the general population. It should be noted that resiliency and coping are related terms and often used interchangeably to illustrate a set of strategies and techniques used by individuals to offset stressors [12, 13, 18].

Previous papers targeting resiliency have historically focused on certain adversities such as exposure to traumatic events and adjustment to physical disease. However, increasingly there has been a push to widen the scope of resiliency research to encompass chronic conditions including disability or autism [8, 15, 17]. As autistic individuals often face unique adversity due to experiencing higher level of stress loads and mental health conditions compared with other neurodivergent people, their ability to gain and maintain resiliency merits exploration [17, 19, 20]. The previous literature primarily focused on only supporting families to remain resilient, rather than the autistic individuals per se. It has been indicated that using social groups to help with problem-solving, cognitive coping and stress management strategies can be considered by parents [14, 21]. Resilient parents are more likely to approach adversity in a positive manner and work with their child in a way which results in meeting and overcoming the demands of adversity [22].

There have been very few studies that have explored the nature of resiliency as it pertains to autistic individuals, making this area one for further exploration. Previous studies found that resiliency amongst autistic individuals may be facilitated by societal attitudes, support systems and the ability to function within their daily and familiar activities [14, 22, 23]. While some research has demonstrated no significant difference in the presentation and impact of resiliency between individuals with ASD and other neurodivergent populations, some literature has indicated that autistic individuals may demonstrate lower levels of resiliency [24–26]. Such increased likelihood of lower resiliency may be attributed to the impact of autistic symptoms or co-occurring conditions on daily stress levels and mental health [24, 25, 27, 28]. However, coping strategies associated with resiliency, such as utilising social supports or mastery, have been shown to offset these outcomes [28].

Given that an increase in the number of chronic stress or adverse life events, especially in childhood, can enhance the possibilities of problematic outcomes later in life, finding effective ways to develop resiliency in autistic individuals is essential [24]. It has been shown that fostering existing internal protective factors, teaching additional resiliency skills such as proper coping methods, and facilitating the implementation of external protective factors by having a better person-environment fit could help mitigate the impacts of adversity and lessen the burden on individuals as well as their families [24, 29–31]. While innate qualities such as such as optimism, sense of humor, and self-esteem have been shown to have an impact on how individuals respond to challenges, the literature suggests teaching the protective factors associated with resiliency such as social skills, ability to cope and emotional regulation could help with resiliency [24, 30]. However, there are few studies that directly explain what these strategies are and how they can be facilitated through perspectives of autistic individuals and their parents.

Thus, there remains gaps in the literature regarding advancing resiliency in autistic individuals. Considering the increased incidence of stress and the adverse physical and psychological impacts of stressors, identifying how we can advance and teach resiliency among autistic individuals merits further research. The current study aims to investigate how resiliency can be advanced among autistic individuals, through perspectives of autistic individuals themselves and their parents. This will help fill gaps in the literature and inform developing programs to enhance resiliency amongst autistic individuals.

## Methods

### Study design

We employed an interpretive description methodological approach, which is an inductive way to understand a phenomenon by valuing participants’ subjective knowledge and generating interpretations that can be applied in clinical practice [31]. To examine the research question, our team gathered qualitative data through in-depth, semi-structured interviews with autistic individuals and parents of autistic individuals.

### Sample

Invitations to participate in the study were sent to clinics across Canada who worked with autistic individuals, posters were put up in local community centers, and snowball sampling was used. During snowball sampling, study participants were asked if they were able to reach out to their networks to help find others to participate in the study. This study looked at two groups of participants: (A) autistic Individuals and (B) parents of autistic individuals, given that autistic adults often rely on their parents well into adulthood. Inclusion for autistic individuals was met if they (a) were 17 years old or older, and (b) a psychologist delivered their diagnosis of autism and participants self-reported their diagnosis during the study. Any parent could be included if at least one of their children had autism. It was required that all participants be able to verbally communicate in English.

In total, 18 participants attended the study. This includes autistic individuals (*n* = 13: 6 males, 7 females), parents (*n* = 5: 1 male, 4 females). Our participants were distributed across 8 Canadian provinces including: Alberta (3), Prince Edward Island (2), Ontario (2), Quebec (3), British Columbia (1), Nova Scotia (3), Manitoba (2), and New Brunswick (2).

The average age of autistic participants fell within the range of from 27 to 53 years, and all were functionally independent and cognitively able to perform their daily activities with no to minimal support. Many of them reported that they had other psychological conditions alongside their autism, with six mentioning attention deficit hyperactivity disorder, three a concurrent learning disability, and three stated a mental health illness. Parent participants fell within the ages of 46 to 63 years. The average age of their autistic child was 29.75 years (SD = 4.57) with the age range being 25 to 36 years old. Some parents reported that their adult child had a co-occurring condition, with one parent indicated their adult child had attention-deficit hyperactivity disorder, another parent reported that their adult child had a learning disability, and two parents noted that their adult children had a mental health disorder.

### Data collection

Participants, who expressed interest in the project, were contacted to attend the interview at the time and location (e.g., face to face, skype, phone, zoom) agreed by participants and interviewer. The data collection was completed between January 2022 to January 2023.

Prior to the interview, participants were asked to sign the consent forms. They were also sent an online form to enter their demographic backgrounds. Each interview conducted for this study lasted for about one hour, was semi-structured, and consisted of open-ended questions. The interviews were conducted by our trained research assistants. All interviews were audio recorded on a digital recorder, and all the files were stored on a password-protected computer. The interview explored the strategies that can be used to build and advance resiliency among autistic people. The interview questions were developed by our team, including two clinicians in the field of autism and one autistic parent who served on our advisory committee from the outset of conducting the project.

At the beginning of each interview, the interviewer asked participants about how they define resiliency to make sure we have a similar concept in mind. Our participants’ definition of resiliency was not different than general populations. Given that the scope of the paper was to understand how to build resiliency among autistic individuals, we included the respective data. During the interview, participants were asked to share if any question may require further explanations. Examples of the questions that were asked include: What environmental and personal factors can help facilitate resiliency in autistic individuals? What strategies should be used to reinforce mental health and resiliency? How can we support autistic individuals to develop resiliency? How do you think we can teach resiliency among those on the spectrum? Compensation was given to each participant of the study in a form of a $30 gift card. This study was approved by University Behavioural Research Ethics Board (#2020–5108) and we followed all the required protocols to maintain the confidentiality and security of the data. We replaced all participants’ names with pseudonyms to keep them anonymous.

### Data analysis

All audio-files of interviews were transcribed verbatim, by a research assistant. Another research assistant double checked the transcript to ensure their accuracy. We used interpretive description approach, which is an inductive and constructive way through epistemological perspectives to co-create knowledge and analyse data [31]. Each transcript was independently coded by two trained research assistants, using NVIVO software. Our research assistants were graduate students with health-related backgrounds during the study. To establish reliability between the two research assistants during thematic analysis, we had several meetings to train them how to code and we compared their codes to resolve any discrepancies. The two research assistants read each transcript several times to find similar patterns in data, prior to coding. They coded each transcript independently line by line. We had regular team meetings to discuss their thoughts about patterns in data and to resolve any disagreements. After completion of codes, we grouped similar chunks of concepts to develop categories and then grouped larger overarching concept to develop themes that could capture the nature of these concepts [31].

To ensure credibility of data, we used reflexivity by writing down our assumptions, personal attributes, qualifications/experience, and biases prior to data interpretations to make sure we are aware of them [33]. None of our research members had relationships with participants. We also used member checking by sharing our interpretations of data among participants to make sure they are reflecting of participants’ thoughts and experiences [33].

## Results

Participants in our study noted three distinct ways to help build resiliency among autistic individuals that consequently can promote their mental health. They discuss several context-based strategies, including those that individuals can use independently, strategies that can be facilitated by community supports, as well as innate factors that may affect one’s capacity to build resiliency (See Table 1).

**Table 1:** Strategies to advance resiliency in autistic individuals

Self-Reliant Strategies	Community Based Facilities	Contextual and Individual Characteristics
Reactive responses Proactive solutions Daily routines	Leisure and activity support Care supports Interpersonal supports	Fixed traits Age Contextual characteristics

### Self-reliant strategies

The first theme discusses the ways in which autistic individuals can be taught to independently enhance their resiliency through reactive responses, proactive solutions, and daily routines.

#### Reactive responses

The majority of participants mentioned that emotional regulation is a strategy that can be used as a form of reactive response to help with coping. A variety of techniques were suggested ranging from letting out aggression in a controlled manner, by for example “*punching pillows to help” (Jason*,* an autistic individual)*, or taking “*5 or 10-minute breaks” (Sami*,* an autistic individual).* However, the most common theme surrounding the construction of successful regulation capacities was that individuals should thoroughly understand what it feels like to be in a regulated state. James, a parent points out that, *“It’s hard to self-regulate and be calm If you don’t know what it feels like to be calm*,* if you’re always agitated.”* Overall, strategies regarding emotional regulation involves what the individual needs in the moment and finding the space to honour those needs.

Once an individual has successfully regulated their emotions, participants noted that they would need to find the means to overcome or “push through” the challenge they were facing. The most common idea suggested by participants was that individuals can overcome challenges with patience. Alice, a parent, suggested that, *“You know*,* just patience*,* you have patience and just take your time [laughs] and keep going.”* In addition, Jason, an autistic individual, suggested that avoiding adverse experiences, such as through ignoring may be helpful in helping to take a break from the problem and solve it at a later date. He says, *“Ignore things*,* block them of your mind. Don’t think about them. Think about something else.”*

Although participants mentioned deliberate strategies that individuals on the spectrum could use to remain resilient in the face of adversity and overcome their challenges, it was clear that there was no “one size fit’s all solution”. Michelle, a parent notes that:

So the better way of approaching that [adversity] is either meeting the needs of each person individually, or giving everyone a kind of better understanding of what to do and… if you ever have these issues, there’s so there’s multiple ways to approach them.

This quote is aligned with ‘no one size fits all’ which implies the heterogeneity of autism. This shows the importance of individualistic strength-based approaches to support the unique and special needs of autistic individuals.

#### Proactive solutions

Participants discussed the idea that autistic individuals must be able to set boundaries to avoid burnout. For example, one parent suggests that, “*It’s so important that the [autistic] individual you’re working with feel that they’re in control and that they’re in charge so they can determine the pace*,* they can determine the flow of information”*. In addition, Tiffany, an autistic individual, mentions that caregivers can also participate in this process, by prompting their child to think about what boundaries mean to them. She states that caregivers should, “*Ask them [autistic children]*,* and to talk to them [autistic children]”*.

Participants urged others to note that it is easy for autistic individuals to lose resiliency, if there are unfair expectations placed on their capacities. Teaching those on the spectrum to *“Just be comfortable with themselves*,* the way they do things” (Calsey*,* parent)*, may help individuals understand that they do not need to push themselves beyond their limits, which could result a loss of resiliency. Viviane, a parent, mentions that caregivers can also avoid setting these expectations for their child, which may contribute to the way they perceive themselves stating that, *“I don’t expect him [my autistic child] to be exactly like all the other kids because that’s not who he is*”.

Participants consistently spoke about the need to teach autistic individuals how to advocate for themselves so that they can avoid adverse experiences that may result in a loss of resiliency. Sami, an autistic individual notes that, “*Self-advocating*,* and Community Advocating*,* advocating is really what has led to my survival in the world and probably to a lot of other peoples on the spectrums*”. James, a parent, points out that before an individual can feel confident advocating for their needs, they must understand that they have the capacity to engage in advocacy. He states that:

I think that one of the ways in which you empower people with disabilities… is that you have to spend a lot of time thinking about…the ability to support the free and reasoned expression of choice.

These quotes imply the importance of advocacy to support autistic individuals with their mental health and wellbeing. Such capacity building helps with readiness and preparedness of autistic individuals to be able to actively and autonomously involve in their decision-making process.

#### Daily routines

The consistency of daily routines was noted as a factor which helped participants to maintain their resiliency. For example, Sami, an autistic adult mentions that “the unknown” often triggers her anxiety, and thus, keeping a set routine helps her to remain resilient. She comments:

Predictability and certainty is what it is right, … Like I find it stressful enough trying to deal with things … As they come along, because then you don’t know what’s up ahead, right?

Participants also mentioned specific hobbies that individuals engage in can enhance their resiliency. For example, Dorian, a parent explains that her son enjoys physical activities to remain resilient because “*he’s [my autistic son is] focused on what he’s seeing in his environment and that physical exertion”*. In contrast, others, such as Jason, an autistic individual, explain that less exertive activities may also be helpful. He says,*“ What do I do? Sleep a lot*,* I like to take naps. I talk to some of my friends. We play video games together. Helps relieve some of the stress.”*

Technology provides a creative, and often irreplaceable, set of tools which can help in building resiliency. Participants mentioned that technology may be able to assist individuals in learning new skills or developing pre-existing skills that enhance resiliency. For example, Jason, an autistic individual, mentions that he uses video games to gain access to a plethora of social interactions, *“while you’re playing video games you are also developing your social skills and talking with other people”.* Others, such as Leslie, a parent, noted that these technologies could be used to develop skills through simulating real world, *“I wonder if we could use something like that [virtual reality] to explain*,* like we’re going to go to the beach and this is what the beach is like”.*

These quotes highlight the application of technology in simulating real environments for individuals on the spectrum to help them have additional opportunities to practice. This implies that by providing daily routines through the aids of technologies, individuals can improve their wellbeing and alleviate their stress levels.

### Community based facilities

Alongside developing independent strategies to enhance resiliency, autistic individuals may utilize external resources in their community to build resiliency, such as by leisure and activity support, care supports, and interpersonal supports.

#### Leisure and activity support

The interactions between individuals in community activities was noted by participants as a unique way for those on the spectrum to build resiliency. For example, Alice, a parent, mentions that through social community groups, her adult child was able to set boundaries and leave conversations when he was uncomfortable. She says, *“I always say*,* let’s give it a try*,* we’ll stay 10 minutes and if it doesn’t work out*,* you [adult child] always have to be willing to leave*,* or willing to adjust to your [needs]*,* or you can go”.* Tiffany, an autistic individual indicates that community groups gave her a familiar space, where she could continuously go to push herself outside of her comfort zone, which helped test her resiliency in a controlled environment. She says, *“I love the idea of being able to practice*,* I do things like I will rehearse conversations in my head and not even in my head sometimes out loud [in community groups]”.* Social programs were mentioned as a space for autistic people to create connections, which helped enhance their resiliency. Dorian, a parent, says: “*When peers have an opportunity to talk about their shared experiences of having autism … they can bond with each other*,* they develop that type of resiliency with guidance and support.”*

Recreational community-based activities were also mentioned by participants as a way to provide individuals on the spectrum a space to broaden their skill sets, to enhance resiliency. For example, a parent mentions that individuals on the spectrum can, “*build some confidence*,* social movements*,* and some achievement whether it can be music or sports”.* Similarly, Jason, an autistic individual, indicates that his participation in community activities showed him how rewarding it can feel to overcome a challenge, *“There are many challenging things we do in community*,* and then when you get them done*,* you feel some sort of that feeling of like you did something*,* accomplished it.”*

Participants were also given a space to suggest community programs which, in an ideal world, would be available to help individuals on the spectrum build resiliency. Many participants expressed the need to teach resiliency though experience. A parent, suggested creating a program that allowed individuals to experience real life scenarios in a controlled setting. She says, *“But if you can practice that [scenario] before*,* you can move past the parts that you do not enjoy and the parts that are there that you like [in real life]”.* A lack of individualised activities was mentioned by participants. For example, Sami an autistic individual notes that she often got discouraged in programs that were not designed for her and thus could not benefit by attending:

*“I would be extremely uncomfortable by joining. Like*,* some kind of like a social dance program*,* and I’ve never really done it before*,* and it’s kind of like…*,* and then seeing everybody else*,* what is it*,* catch on faster.”*

#### Care supports

With many supports available, participants mentioned the ways in which they were aided by service providers. Our participants noted that an outside voice was often helpful in helping them contextualize the potential barriers they might be facing and providing a fresh perspective on ways to overcome them. Alice, a parent, mentions, *“It’s easy to overlook things when you’re a parent*,* but when you’re the service provider sometimes the answers are so obvious”.* Service providers were also praised by participants for their ability to form partnerships with autistic individuals and to work with them to advance resiliency in accordance with their goals and values. James, a parent, mentions, *“There’s got to be relationship*,* because you [service providers] have to be able to change what you’re doing.”* This shows the importance of the ability of service providers to recognize, overcome and adapt to autistic specific challenges to help build resiliency. Similarly, Luke, an autistic individual, draws on his experience to ask service providers, “*Do not get angry and frustrated with ASD people*,* but to use like certain interactions as learning experiences for how to deal with them basically”.*

The role of family and close networks in helping autistic individuals to foster their resiliency is pivotal. Parents emphasized that for them to actively engage in advancing resiliency of their children, they too were required to remain resilient. Molly, a parent, mentions that respite could be used as a tool to help caregivers relax and reset, *“So it is important to use the money that you get from the government for respite”.*

Participated indicated that mental health was a key factor in determining an individual’s capacity to build resiliency. The role of service providers in improving this facility was emphasized. It was mentioned by participants such as Luke, an autistic individual, that the therapy was a good way to ensure that his capacities did not deteriorate to an extreme when experiencing a mental health crisis,*.* He says, “*I have my own mechanisms too. I mean*,* I seek counseling as well just to keep my head above water”.* Later, Luke stated that counselling could be used to help with his independence. He says, “*going to all those different services help me being able to figure out how to do stuff more independently*,* than always having to rely on other people to help fix them for me”.*

#### Interpersonal supports

Although professional community support was often required to help individuals on the spectrum foster resiliency, the importance of family and friends was also emphasized. Because of their daily presence in the lives of individuals on the spectrum, family members relayed the unique strategies they used to support resiliency building. Tiffany, an autistic individual, mentions that parents can try to use everyday moments as a time to teach, *“Now*,* you can break down that skill*,* and say*,* hey*,* this is what we’re doing*,* you know*,* it’s sort of like those teachable moments”.* Others noted that family may play an imperative role in resiliency building because of their ability to more easily interpret the needs of autistic individuals. To this point, a parent, says, *“Some individuals are much more comfortable [with other people]*,* including a particular caregiver or a family member who can sometimes interpret information”.*

Peer networks were also another support system emphasized by participants. Madeline, a parent, suggest a strategy to autistic individuals who often find themselves excluded from social circles. She states, *“have a group of people that you can trust*,* and go confidently to talk to*,* it makes like a world of difference”*. Another parent indicates that “*We learn from peers”.* These show that close networks are important for autistic individuals when trying to build resiliency as the individuals within them can act as role models.

### Contextual and individual characteristics

The theme contextual and individual characteristics discusses how one’s personal characteristics can affect their ability to build resiliency. These factors include, fixed traits, age, and contextual characteristics.

#### Fixed traits

Participants recognized how the innate characteristics of those on the spectrum may affect their capacity to advance resiliency. For example, Alyssa, a parent, mentions that it may be difficult for her son, who is non-verbal, to explain when he has reached his limits, and therefore he is more likely to burn out. She says, *“When we work on communication more*,* he’ll [my autistic son will] be able to communicate with me*,* but when he’s starting to feel stressed out*,* [he is] not able to handle it.”* Likewise, another parent explains that the minimal use of facial expressions, a characteristic common of those on the spectrum, may also impact communication, and result in burnout. He says, *“Because a lot of autistic people don’t show it [signs] in their facial expressions. So*,* people can’t tell when you’re stressed out.”*

#### Age

Participants noted that age was an important characteristic to consider when deciding when and how to teach resiliency. For example, Calsey, a parent, notes that her son has not yet developed the ability to recognize situations in which he may need to be resilient. She mentions, *“I don’t think he’s [my son is] too bothered by people*,* like [others] maybe thinking he’s strange.”* Others, such as Tiffany, an autistic individual, stresses the unique process of teaching resiliency when people are still young:

When you’re a teenager, you’re going through so many changes and really struggling to find yourself. And at that point, it could be so easy to be held back by your own insecurities and to feel like, if you don’t have that resiliency to gain, just getting to normalize being at the bottom.

This shows the importance of age in developing resiliency, when dealing with stressful situations among autistic individuals. A question that naturally followed after discussing the effect of age on resiliency was, *at what age should resiliency be taught?* Although one parent stated that *“I don’t think it’s ever too late”* to start learning how to become resilient, the most participants mentioned that teaching Resiliency would be most effective when taught to younger individuals. Tiffany, an autistic individual, suggests that the reason for this may be because:

If we teach them [resiliency skills]…, start from very little, preschool, if that just becomes a normal way of believing in yourself and handling yourself and knowing that problems are going to exist, everyone has problems, but this is how we get through them.

#### Contextual characteristics

Participants noted that the resiliency of those on the spectrum was often influenced by dynamic factors such as health. A common co-occurring condition associated with autism is the high rates of psychiatric disorders. These can often compete with other cognitive functions. As Madeline, a parent, points out, when someone is experiencing a several mental illnesses, it can often be difficult to teach them resiliency, “*Being able to cope with that kind of stuff is insanely hard when you got a thousand different things going on in your mind at once”.* This quote stresses the importance of ensuring that individuals are mentally prepared to engage in resiliency training before the commencement of a program.

Participants also mentioned the impact of physical health on emotional wellbeing and resiliency capacity. Alex, an autistic individual, notes that physical health deficiencies can result in a wide array of consequences and often affects one’s ability to learn resiliency:

I mean it starts with neurophysiology, our whole body, if your sympathetic system is always activated a lot of things go from, you know, I mean you can have brain fog, you could have cardiovascular respiratory disease, your immune system is compromised, like I mean there’s just a wide array of things that can happen.

Participants explained that often, the extra emotional and physical burden experienced by autistic individuals result in lifelong impacts. For example, Alex, an autistic individual, compares the toll of autism to that of AIDS, noting that this impacts one’s ability to be resilient. He says, *“Yeah I think it can holistically impact a lot of areas in our lives*,* I mean just look at the AIDS study*,* there’s repercussions throughout a lifetime.”* Tiffany, an autistic individual, specifies that, for example, one of these lifelong effects may be that individuals on the spectrum naturally lean towards a mindset of pre-emptive defeat when trying to solve a problem. She says, *“When you don’t have that resiliency to get back up*,* you stay down*,* and then you just get used to being down. That’s really a crappy place to be.”*

## Discussion

Few studies have explored the concept of resiliency from the perspective of autistic individuals. However, the current paper provides a unique perspective as it is one of the few that focuses on “how to build” resiliency by describing specific supportive strategies. The current study shows that individuals can consider self-reliant strategies, community based facilities, and contextual and individual characteristics to advance their resiliency. The utility of these strategies is context-based, which depends on the individual’s readiness, their preparedness, and available services. However, addressing the perceived challenges or deficits experienced by individuals with autism should not only be relied on strategies employed by individuals per se. This may place greater pressure on these people without considering strategies to inform and educate the wider population.

It should be noted that resiliency is a crucial element in promoting a strong mental health framework [12, 13]. Given the lifelong nature of autism, it is important that those on the spectrum can enhance their resiliency to overcome challenges and reach their full potential. As autistic individuals are specifically prone to experiencing anxiety and stress, it is important to understand what services and strategies can be most effectively used to enhance their resiliency [34]. Models such as the social ecological model of resiliency or the social model of disability suggest that social context and society both play a key role in determining how an individual responds in the face of adversity and how external factors can help them [35]. Because of this complex relationship between resiliency and external factors, it is important that the viewpoints of individuals on the spectrum be deeply investigated to further understand the necessary nuances of the situation and to properly inform future policies [35–37].

One of the major strategies discussed by our participants was utilizing community resources. Such supports could be formal programs offered by institutions such as organized sports or respite, or they could be more informal such as through friendships and family. Interpersonal connections can be considered as a way to enhance resiliency through increasing sense of belonging. This aligns with the social model of disability, which suggests that individuals within society can determine how disability should be perceived [38]. Furthermore, through observing and modeling behaviours early on, autistic individuals can learn how to behave in certain situations and demonstrate resiliency. Formal programs and community supports were also described as a good way for individuals on the spectrum to enhance resiliency by engaging in social interactions and gaining new skills and becoming empowered. Although our study suggests that formal and informal community supports may help to enhance resiliency, systemic barriers such as lack of funding or long wait times may limit service access [39]. This suggests that policy makers and service providers should not only work on implementing new programs to enhance community support for autism, but in addition, they should try to facilitate the access [39].

Participants also highlighted strategies to proactively enhance resiliency as well as how to reactively utilize resiliency in the face of adversity. Proactive solutions mainly centered around the idea of finding ways to manage adverse experiences that would test one’s resiliency in the first place. Much of this work centered around self-advocacy strategies and how to remain “within your limits”. Although such strategies are mainly perceived to be used by individuals, it should be noted that the requirement to self-advocate is primarily due to existing issues in service access [40, 41]. Therefore, it is important to understand perspectives of autistic individuals about effective services.

Participants also discussed how reactive strategies could be employed to help individuals cope with a long- or short-term momentary lapse in resiliency. Participants suggested strategies which helped to regulate their emotions, such as taking a break or engaging in physical activity. The suggested approaches mainly involved adaptive or psychologically mature responses as opposed to maladaptive, immature responses [42]. Psychologically immature responses may consist of acting out or being passive aggressive, whereas mature response may look like dealing with one’s emotions through planning to ease discomfort [42]. The difference between mature and immature response is important to highlight as psychologically immature responses may lead to additional burnout and contribute to the loss of resiliency [43].

Although strategies to enhance resiliency were suggested by stakeholders, our participants noted that individual characteristics of those on the spectrum including innate attributes of social traits, age, and health conditions could influence their resiliency. These attributes can not only affect how individuals engage in self-reliant strategies, but in addition, how they engage in community supports. Previous literature indicated the effect of gender or age on the ability of an autistic individual to deal with adverse situations [44]. However, as a novel finding, participants in this study underscored the impact of age on the ability of autistic individuals to gain and learn resiliency. We found that younger individuals may not be as aware of the factors that could serve to challenge their resiliency such as societal expectations. This may be consistent with the social ecological model of disability which describes how context can influence a response [35]. Participants also highlighted that as one ages, they need to deal with a plethora of anxiety-provoking situations that utilize their mental and cognitive capacities. With their capacities under greater strain, it becomes much more difficult to remain proactively and reactively resilient [45].

The literature also touches on how individual characteristics can affect access to community resources both directly and indirectly [46]. For example, although many community-support programs for youth on the spectrum are accessed through school [47], adults on the spectrum may find it difficult to engage in programs that help to enhance their resiliency because of age limits. Furthermore, co-occurring conditions may indirectly affect access to external resources, because individuals may not physically or mentally be able to participate in available programs [48, 49]. Alternatively, restrictive or repetitive behaviours can create barriers to service access, because they may result in increased social isolation, which is negatively correlated to community involvement [47]. Regardless of how co-occuring conditions create barriers in accessing to services, and in accordance with the social model of disability, these challenges illustrate a gap in the system which has unsuccessfully met the individual needs of autistic communities [38].

### Limitations and future directions

Although this paper adds to the literature about how to build resiliency among autistic individuals, it has several imitations. First, the sample size of the project was small, which limits how it represents the diversity of autistic individuals, affecting the transferability of our data to other contexts. Our sample included autistic adults who were verbal with low support needs, so the perspectives of those who are non-verbal or with higher support needs are still unknown. Although we did not find any differences in responses of parents and autistic adults, this may also be due to the sample size. Second, we only involved autistic individuals and their parents to collect their inputs. However, involving other family members of autistic individuals such as their siblings or partners may be considered in future studies to better highlight the resiliency strategies. Third, as the scope of the project was how to advance resiliency among autistic individuals in the context of mental health, it would have been beneficial to collect more comprehensive demographic information about mental health symptoms, daily activities, and support services that autistic individuals used, their ethnicity, and whether their parents were on the spectrum or not. Given that each strategy is context-specific, this information can be included in the future studies to help with interpretations of what strategies can be used in which context.

## Conclusion

The findings of this study contribute to the literature by proving both specific strategies to build resiliency for individuals on the spectrum through the perspectives of autistic people and their parents. Previous literature primarily discusses the resiliency of families, while this study focuses on methods to enhance resiliency within the autistic individuals themselves. This study provides novel insights into methods in which resiliency can be achieved for individuals on the spectrum in the context of mental health and may inform future efforts of service providers, policy makers, and advocates.

### Electronic supplementary material

Below is the link to the electronic supplementary material.

Supplementary Material 1

## Data Availability

No datasets were generated and the relevant data has been included in the manuscript.
