# Supplementary material for: How to build resiliency in autistic individuals: an implication to advance mental health
Source: BMC Psychol. 2024 Aug 1;12:420. doi: 10.1186/s40359-024-01916-1 (PMC11295548; doi:10.1186/s40359-024-01916-1)
Supplement: Supplementary file 1 — Supplementary Material 1 [file 40359_2024_1916_MOESM1_ESM.docx]

Interview guide sample questions:

1. What environmental and personal factors can help facilitate resilience in autistic individuals?
2. What strategies should be used to reinforce mental health and resilience?
3. How can we support autistic individuals to develop resilience?
4. How do you think we can teach resilience among autistic people?
5. Are there any barriers in developing resilience?
